# Brazilian Bimodal Bilinguals as Heritage Signers

**DOI:** 10.3390/languages3030032

**Published:** 2018-08-10

**Authors:** Ronice Müller de Quadros, Diane Lillo-Martin

**Affiliations:** 1 Departamento de Libras, Centro de Comunicação e Expressão, Universidade Federal de Santa Catarina, R. Eng. Agronômico Andrei Cristian Ferreira s/n, Trindade, Florianópolis SC 88040-900, Brazil; 2 Department of Linguistics, University of Connecticut, 365 Fairfield Way, Storrs, CT 06269-1145, USA

**Keywords:** bimodal, bilingual, heritage language, sign language, code-blending, Brazilian Sign Language, Brazilian Portuguese

## Abstract

This paper presents an analysis of heritage signers: bimodal bilinguals, who are adult hearing children of Deaf parents who acquired sign language at home with their parents and the spoken language from the surrounding community. Analyzing heritage language with bimodal bilinguals who possess pairs of languages in different modalities provides a new kind of evidence for understanding the heritage language phenomenon as well as for theoretical issues regarding human language. Language production data were collected from four Brazilian bimodal bilinguals separately in both sign and speech, as well as from monolingual comparison Deaf signers and hearing speakers. The data were subsequently analyzed for various grammatical components. As with other types of heritage speakers, we observed a great degree of individual variation in the sign (heritage) language of balanced participants who patterned similarly to the monolingual signers, compared to those whose use of sign language differed greatly from monolinguals. One participant showed some weaknesses in the second (spoken) language. We approach the variation in language fluency in the two languages by considering the different contexts of language development and continuing use.

## 1. Introduction

### Heritage language: The Case of Hearing Children of Deaf Parents

This paper focuses on the language use of hearing adult children of Deaf^1^ parents in Brazil. Children of Deaf adults—known as Codas—who are not deaf themselves may be said to acquire a sign language as a heritage language (Compton 2014; Palmer 2015; Pichler et al. 2017; Reynolds 2016). A heritage language is a minority language used in a specific socio-cultural context in which a different language is dominant in the community. Benmamoun et al. (2013, p. 132) define “heritage speakers” as, typically, second-generation immigrants who live in bilingual contexts. In this case, the heritage language is the language the children are first exposed to at home, before subsequently becoming dominant in the language of the broader society in which they live. In Brazil, for instance, where Brazilian Portuguese (BP) is the dominant societal language, any other language used in a specific community may be considered a heritage language including immigrant, ethnic and sign language communities. Similarly, in the United States, immigrant (and colonial) languages, as well as ethnic (Fishman 2001) and sign languages, are considered heritage languages in an English-dominant bilingual context. Because of the nature of their language acquisition, heritage speakers are often more competent speakers of their L2 (the socially dominant language) than their L1 (heritage language). The imperfect control that heritage speakers exhibit over their L1 often differs in significant and consistent ways from the imperfect control of second-language learners; frequently, heritage learners are understood to experience ‘attrition’ of their original language system, rather than incomplete acquisition (Polinsky 2006; Scontras et al. 2015).

Heritage speakers can shed useful light upon the current theoretical discussion about the nature of language, allowing us to adopt a novel approach to Chomsky (1986) important question: “what do we know when we know a language?”. We may have intuitions about what a native speaker knows with respect to their language, but heritage language forces us to consider more deeply the question of what exactly it means for a person to be a native speaker. There is a consensus that native speakers/signers differ from non-native speakers/signers of a language because they acquired their language from a very early age within a natural input environment—this makes native speakers different from L2 speakers but identical to heritage speakers. Heritage speakers, like native speakers, acquire a home language naturally at an early age—the difference is that they also acquire a large community language at the same time, which they gradually come to rely on as their primary language in the dominant society (for example, Chinese at home and English at school and elsewhere).

Codas follow the model of heritage language acquisition when acquiring their sign and spoken language: they acquire sign language at home with their parents (and, in some cases, with the broader Deaf community) and the spoken language of their country with other people (hearing family members, colleagues at school, neighbors and other hearing people). In some cases, this acquisition model can lead to a non-native mastery of sign language in adulthood. We will follow Emmorey et al. (2008) in referring to heritage speakers of sign language as ‘bimodal bilinguals’, because they have two languages in two different modalities (sign and speech). Applying Benmamoun et al. (2013, p. 133) definition of heritage speakers to bimodal bilinguals, bimodal bilinguals are early bilinguals who grew up seeing (and signing) the heritage language (L1) and hearing (and speaking) the majority language either simultaneously or sequentially in early childhood (that is, roughly up to age 5) but for whom the majority language became the primary language at some point during childhood (at, around, or after the onset of schooling). As a result of language shift, by early adulthood a bimodal bilingual may be strongly dominant in the majority language (spoken language), while the heritage language (sign language) will now be the weaker language.

Although in Benmamoun et al. (2013) definition the heritage language necessarily becomes the speaker’s weaker language over the course of time, in this paper, we will use the term “heritage speaker” to refer to any person whose home language differs from the dominant language in the general society. As our discussion below will show, heritage speakers of a sign language may have weak mastery of their L1, or they may pattern more like balanced bilinguals (see Pizer 2008). Lillo-Martin et al. (2014) observe that there is considerable variability among Codas in terms of the balance between the languages they are acquiring. This variation can be attributed both to the amount of spoken language used in the home (some Deaf parents may produce the spoken language vocally, while others do not—though they may follow parts of spoken language through speechreading—and most have knowledge of the written language, although this can vary in different locations) and the degree of support the children receive for their signing. Pichler et al. (2014) conclude that balanced bilingualism is most readily achieved by Deaf families who take time to encourage their children to sign with Deaf people in different contexts, as the wider society does not value sign language. Schools and the surrounding spoken language environment push these children to use English much more than their heritage language. For heritage signers, as for speakers of other heritage languages, the attitude of the children’s input providers plays a role in their language choice (see (Döpke 1992) and (Lanza 1997) for unimodal bilinguals; (van den Bogaerde and Baker 2009) for Nederlandse Gebarentaal (NGT) [Sign Language of the Netherlands] and Dutch bimodal bilinguals; (Kanto et al. 2013) for Finnish Sign Language (FinSL) and Finnish bimodal bilinguals). Over time, however, in general the sign language tends to become the weaker language as bimodal bilinguals begin to privilege the spoken language, even with Deaf interlocutors (Peyton et al. 2001; Kondo-Brown 2006).

On the other hand, since sign and spoken languages use different articulators, bimodal bilinguals differ uniquely from other heritage speakers in being able to produce both their languages simultaneously, using what is called code-blending (Emmorey et al. 2008). Code-blending differs from code-switching in that both languages are produced at the same time—whereas unimodal bilinguals have to learn to suppress one of their languages even when they code-switch, bimodal bilinguals can simultaneously use grammatical knowledge and lexical items from both languages, separately or combined, while continuing to observe language constraints (Lillo-Martin et al. 2014, p. 13). Lillo-Martin et al. (2014) observed that sociolinguistic factors influence the use of code-blending by young bimodal Codas, in the sense that they decide whether to use or avoid blending depending on who they are conversing with. On the other hand, Pyers and Emmorey (2008) found that adult Codas tend to employ grammatical sign-language facial expressions even when conversing in a speech-only modality with monolingual speaking people.

Another intriguing manifestation of bimodal bilingual production is known as bimodal “whispering”. Petroj et al. (2014) describe whispering—in the case of bimodal bilinguals—as the use of the lexical items of a spoken language produced with little or no vibrations of the vocal cords during signing. The authors suggest that this practice serves to reduce the pressure of suppression of the spoken language. The authors found that the American Sign Language (ASL) and English bimodal bilinguals that they studied, whom they described as balanced bilingual language users, accommodated the grammar of their whispering to ASL structure rather than to English structure. Conversely, in contexts where heritage signers have begun to rely on the spoken language as their primary language, we might expect their “whispering” practice to take on more characteristics of the spoken language structure, rather than remaining true to the sign language structure.

All of the studies discussed above give us a good general insight into the linguistic behavior of bimodal bilinguals. The more general heritage language literature, however, provides some important models for further work. In unimodal heritage language, as in bimodal heritage language, significant variation is reported across individuals (see Benmamoun et al. 2013; and Scontras et al. 2015 for discussion). Polinsky (2008), for instance, focuses on noun categorization as a means to investigate both lexical access and sentence processing among American Russian heritage speakers. She argues for the value of noun categorization as a diagnostic tool because it bootstraps morphology, phonology, syntax, and simple semantic structures at different levels of linguistic representation. Similarly, Benmamoun et al. (2013, p. 136), following Polinsky (2005, 2006), advocate for the use of lexical proficiency as a fluency diagnostic. Polinsky (2006, p. 252) found that lexical proficiency can provide a good idea of the structural knowledge and overall competence that a heritage speaker has with each of their languages. In particular, she argues that lexical proficiency scores can be used as a basis on which to characterize the linguistic system of incomplete learners. Using this diagnostic, Polinsky (2005, 2006) found a strong correlation between a speaker’s comprehension (measured in terms of oral translation of a basic word list) and grammatical phenomena (such as agreement, case marking, aspectual and temporal marking, pro-drop, co-reference, and embedding).

In a study investigating these same grammatical areas, Montrul (2011) found a tendency toward the simplification and over-regularization of complex morphological patterns in heritage language, as well as restricted word order. Likewise, Albirini et al. (2013) report that heritage speakers demonstrate significant syntactic vulnerability in their L1. The authors found that heritage language subject-verb agreement morphology is maintained more robustly than noun-adjective morphology in heritage speakers’ oral production, while the unmarked singular masculine is more robust than other categories. These results suggest that asymmetries in heritage speech may be explained by a complex interaction of linguistic areas and frequency factors.

Another study from Albirini et al. (2011) analyzed the syntactic and morphological features (word order, use of null subjects, selection of prepositions, agreement, and possession) of oral narratives produced by heritage Egyptian and Palestinian Arabic speakers in the U.S., paying particular attention to possible morphological gaps and the use of code-switching. The authors identified many non-native trends in heritage speakers’ narratives, especially with respect to the use of plural and feminine nouns. In general, the speakers they analyzed were unable to apply agreement appropriately in overt or null pronominal contexts, indicating that these speakers knew the agreement morphology but lacked control over gender and number placement. The authors also found problems with number formation and agreement and the use of prepositions and possessives. All these gaps involve transfer from the dominant language to the heritage language, indicating incomplete knowledge or attrition of the L1. In terms of code-switching, Albirini et al. (2011) found that nouns were the most frequently switched category, followed by adjectives, then verbs, prepositions and adverbs. The authors concluded that the use of code-switching did not interfere with the basic sentence structure used by heritage speakers and thus did not result in ungrammatical sentences. These results are particularly interesting for our study, which includes the issue of code-blending amongst bimodal bilinguals. As we will discuss in the next section, the “Synthesis” model of language that we adopt predicts that code-mixing (switching or blending) can only felicitously apply when all feature checking in both languages is satisfied.

The goal of the present study is to expand our understanding of bimodal bilingual variation in language fluency in heritage signers’ first language (sign language) and second language (spoken language).^2^ Analyzing heritage language use among bimodal bilinguals who possess pairs of languages in different modalities (a sign and a spoken language) can shed new light on our understanding of heritage language phenomena more generally. The following questions are addressed by the study presented here: (i) What determines the range of variation within the heritage speaker cohort that we are considering? What is the role of language use in a given context? In considering these questions, we will take into consideration the role of sociocultural factors in the development and maintenance of the heritage sign language as well as factors of upbringing and early experience; (ii) What areas are vulnerable—that is, prone to cross-linguistic influence or errors—in both languages and what areas are robust? Why?

## 2. Materials and Methods

To address the research questions, we gathered data in both sign-only and speech-only contexts separately. For each language context, we assessed the following grammatical areas:

- mean length of utterance;
- word-per-minute rate;
- distribution of word types used;
- types of syntactic constructions used;
- verbal morphology production accuracy.

The diagnostic of word-per-minute output rate in spontaneous production is recommended in Benmamoun et al. (2013), following Polinsky (2008). Polinsky (2008) research has found that a heritage speaker’s speech rate may be as low as 30% of the speech rate of full speakers of the same language. Polinsky (2008) also found a correlation between speech rate and grammatical knowledge (lower proficiency speakers have more difficulty accessing lexical items). In our study, we analyzed in detail the participants’ word-per-minute output in each language, as well as their mean length of utterance (MLU) for each output modality. This information was compared with the participants’ sign and speech proficiency scores.

Considering the correlation between word-per-minute rate and lexical proficiency reported in previous studies, we next checked for morphological asymmetries in the bimodal bilinguals’ heritage language. Benmamoun et al. (2013) found that morphological deficits in heritage speakers occur more regularly in nominal morphology than in verbal morphology; they further showed that, within the realm of verbal morphology, agreement is the most vulnerable category while tense is the most robust (reported as well by Montrul (2011)). The authors hypothesize that heritage speakers preserve the mechanisms to generate syntactic structure but have less capacity to preserve post-syntactic operations (and therefore have difficulty mapping from syntax to Phonological Form (PF)). We predict that less-balanced heritage signers will exhibit “errors” in post-computation (phonological and morphological) materials in their heritage (sign) language, when compared to their speech production. If our findings align with those of previous researchers, these errors should occur mainly in verbal agreement. By pulling together all these elements from bimodal bilingual data, our study offers new insight into the rules and constraints that govern heritage language development and interaction.

### 2.1. Participants^3^

The primary participants are four heritage signers from Brazil, who are bilingual in Libras (Brazilian Sign Language) and BP. NT and JB are siblings; JB is the older child and they have a deaf mother and a hearing father. NT and JB’s mother has a Deaf family with a brother, sisters, and cousins who are deaf. She is not formally educated, yet signs in a typical fashion with her deaf family and her children. NT and JB only sign with their mother and they typically do not blend sign and speech since their mother is monolingual in Libras. CL is a sign language interpreter; both of her parents are Deaf. CL’s mother is sister to NT and JB’s mother. Both of CL’s parents had typical primary education. MR is a teacher of Deaf children; her parents are Deaf and they had a typical primary education.

Table 1 summarizes information about the participants. A Brazilian native bimodal bilingual assigned ratings of each participant’s overall fluency on a scale from 1 (low)–7 (high) in Sign (Libras) and Speech (BP). The participants are listed in their order of sign proficiency. For comparison, four Deaf signers of Libras and four hearing non-signers also participated.

### 2.2. Procedure

Data collection for the primary (Bimodal bilingual) participants took place within a larger study of bimodal bilingualism, the Development of Bimodal Bilingualism project (see bibibi.uconn.edu). The comparison data were collected separately, in a similar manner. For the current study, participants watched a short wordless animated movie clip (2 min) and immediately thereafter told the story they had seen to either a Deaf person (for Bimodal bilinguals in the Sign session and for Deaf participants) or a hearing person (for Bimodal bilinguals in the Speech session and for hearing non-signers). The participants were aware of the need to use Libras with the Deaf interlocutor and of the expectation that they would use BP with the hearing interlocutor. All sessions were video-recorded for later analysis.

After the videos were recorded, the processing and coding took place in the lab in several steps. The first step was to produce a complete transcription of the narrative produced by the participant, using the software program ELAN (Max Planck Institute for Psycholinguistics, The Language Archive, Nijmegen, The Netherlands, https://tla.mpi.nl/tools/tla-tools/elan/; Crasborn and Sloetjes 2008). Signs from Libras are annotated using SignIDs, approximate BP translation equivalents written as uppercase glosses (Johnston 1991), with additional conventions for systematic representation of signed utterances (see (Pichler et al. 2010) for further information about the conventions). Each sign is represented in an individual annotation, using separate tiers for the right hand and the left hand. Spoken words are also entered individually on a tier for that purpose. Finally, a free translation is written on another tier, using both the signs and speech as well as contextual information to fill in any gaps in the utterances; the free translation also indicates the length and linguistic contents of each utterance.

Following the annotation process, each session was coded for the linguistic dimensions of interest in this study. We calculated each participant’s production speed in spoken words or signs per minute using the individual annotations to indicate the number of words used. We also calculated the MLU in words (MLUw) in each language using the individual annotations to determine the number of words and the free translations to mark off utterances.

The final stage of analysis added a tier for the lexical category of each individual sign or word (verb, noun, functional category, adverb, pronoun, adjective, or WH-word) and a tier for the syntactic type (declarative, relative clause, embedded clause, coordinate clause, yes/no question, WH-question, fragment, or other). For the bimodal bilinguals, we also checked each signed verb for its morphological accuracy.

## 3. Results

The results of our calculation of production speed (signed or spoken words/minute), and the speech: the sign ratio of production speed is presented in Table 2. This table also presents the results of our calculation of MLUw for both sign and speech. Finally, for the bimodal bilinguals, the table provides the percentage of verbs produced with ungrammatical morphology in Libras.

**Figure 1.**
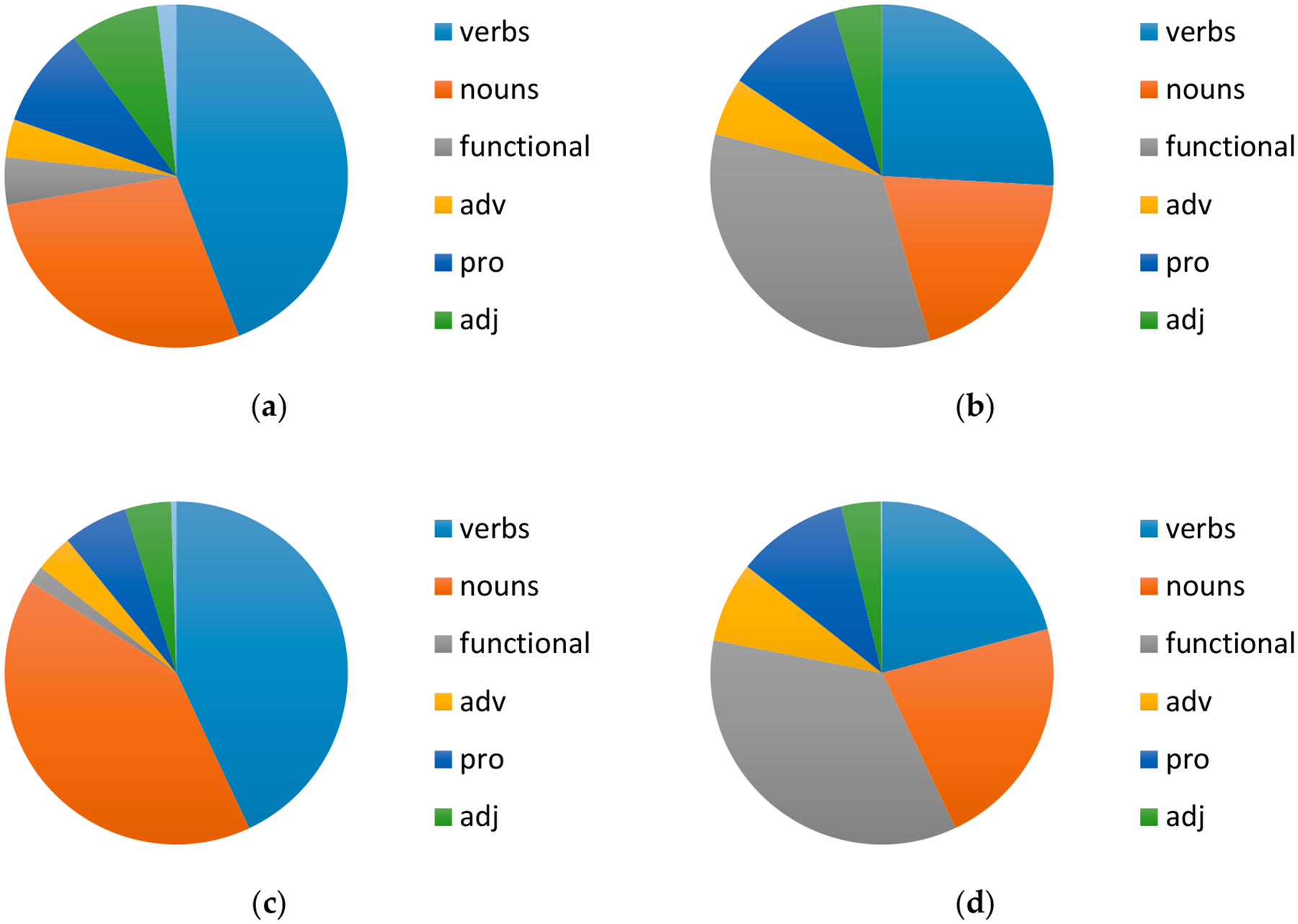
Lexical types used by (**a**) bimodal bilinguals in sign; (**b**) bimodal bilinguals in speech; (**c**) Deaf signers in sign; (**d**) hearing non-signers in speech.

The results of our analyses of lexical categories used in sign and speech are given in Figure 1. We found that the different participants within a group showed a similar distribution within a language, so we have collapsed the results by group/language.

**Figure 2.**
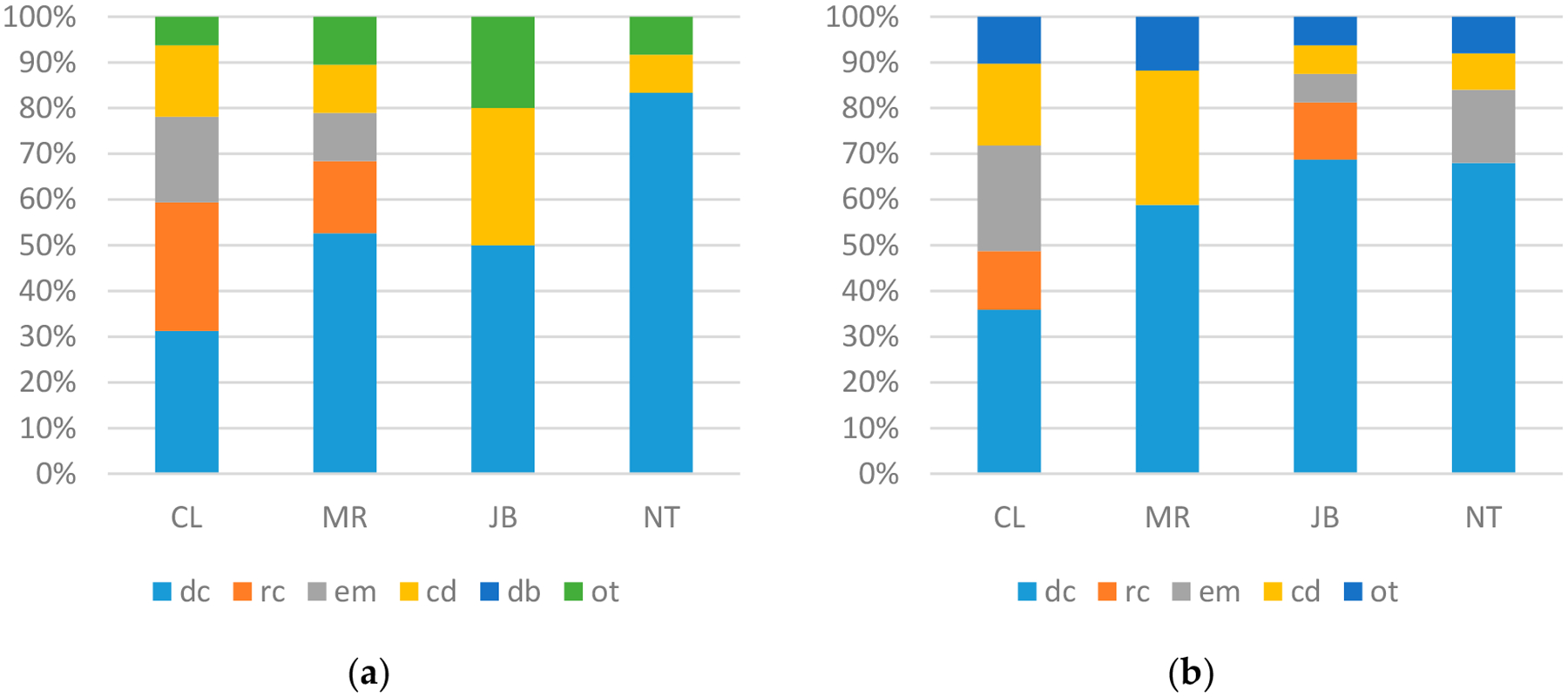

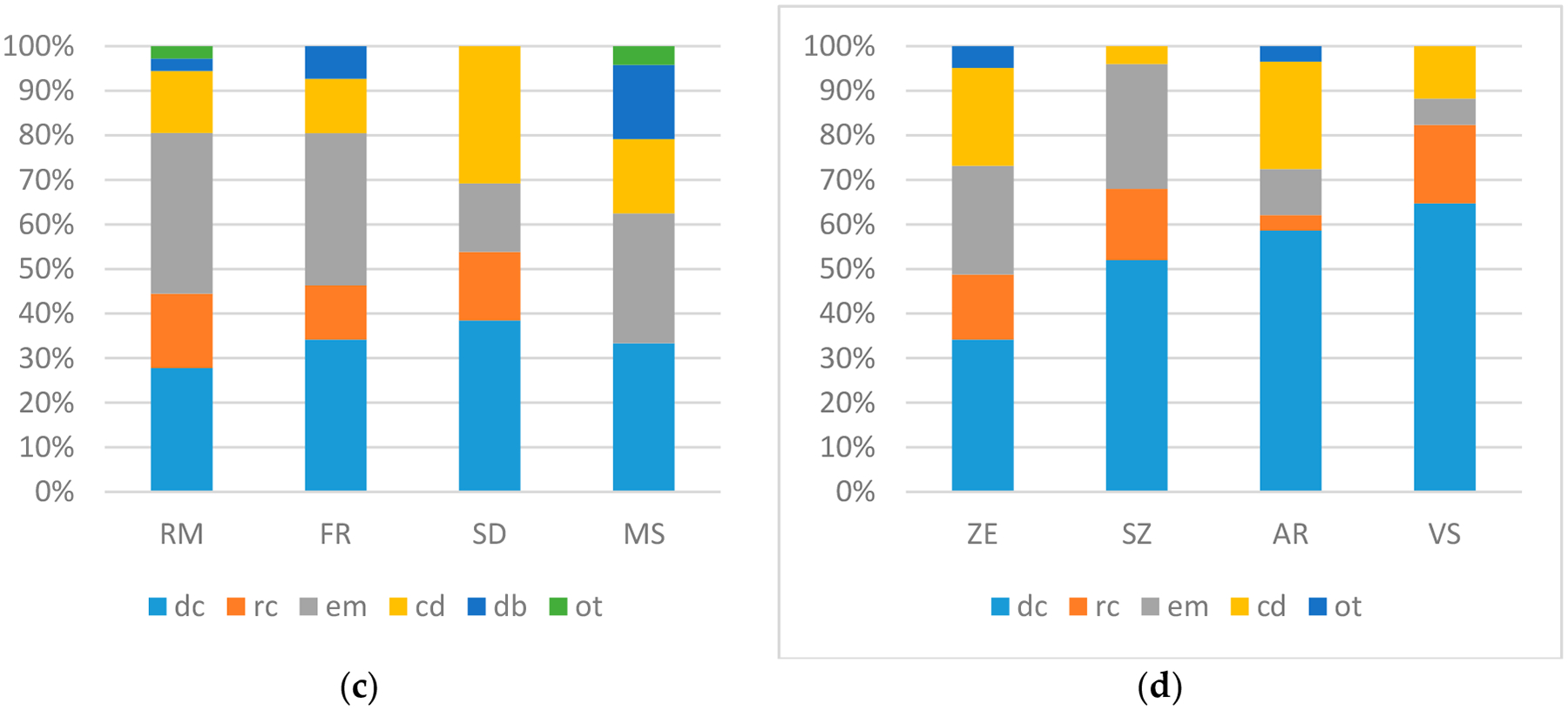
Syntactic types used by (**a**) bimodal bilinguals in sign; (**b**) bimodal bilinguals in speech; (**c**) Deaf signers in sign; (**d**) hearing non-signers in speech. dc: declarative; rc: relative clause; em: embedded; cd = coordination; db: double; ot: other).

The results of our analyses of syntactic types used in sign and speech are given in Figure 2. We found that the different participants within a group showed very different distributions within a language, so we have provided individual participant responses for each group/language.

## 4. Discussion

### 4.1. Empirical Discussion

Overall, the results clearly show a wide variability in proficiency with the heritage language for bimodal bilinguals. This is consistent with a number of studies discussing variable fluency in the sign language across bimodal bilinguals, including Compton (2014); Pichler et al. (2017); de Quadros (2017); de Quadros et al. (2016a, 2016b). The variability in the current data likely stems from individual differences, considering the differences in each participant’s relationship with their two languages. CL, who is the highest-scoring signer and a sign language interpreter, displayed the highest MLUw in both speech and sign, and she produced sign and speech at equivalent rates. MR had a much higher word/minute rate in speech—possibly indicating that Portuguese is her strongest language, even though her signing is good—likely related to the fact that she is a teacher. JB had a lower MLUw and a slower production speed in sign (her weaker language) as compared to speech. NT exhibited the lowest MLUw and production speed in both sign and speech; however, it appeared that the test context made him somewhat uncomfortable, so this might not reflect his usual performance ability. In comparison, his sibling JB was much more comfortable when she did the test in Portuguese.

In comparison to the bimodal bilinguals, the monolingual Deaf participants showed the same or a higher rate of signs per minute and a consistently higher rate of signed MLUw. Only CL, the bimodal participant with the highest sign rating, showed an MLUw close to the minimal range of the Deaf signers. In speech, the monolinguals showed a high rate of speed comparable to MR and JB, while CL and NT were much slower. The speech of the bimodal bilinguals, except for NT, was within the wide range of MLUw in the Portuguese monolinguals. We note that the speech: sign production speed ratio for MR and JB indicates much higher rates of speech; on the other hand, the ratio is balanced for CL, who is strong in both languages, and for NT, who shows weaknesses in both languages. Given the overall lower performance by NT, it might be that, unlike the other participants, he shows the effects of having grown up in a heritage language context in both his languages, something which is also found for some hearing/speaking heritage speakers, with lower rates in both of their languages, as reported by Albirini et al. (2011, 2013); Montrul (2011); Polinsky (2006, 2008).

As for the lexical category analysis (Figure 1), we found that, for the most part, the individual participants did not differ within a language; rather, the greatest differences are observed across languages. All bimodal bilingual participants used a much greater proportion of verbs than nouns in their sign but the monolingual signers showed this contrast less, with two individuals keeping the pattern and two going the other way. The predominance of verbs likely follows from the fact that, in Libras, as a null argument language (de Quadros 1995), it is very common for an utterance to contain only verbs, once referents have been introduced. In the experiment, the story being told centered around a particular referent that, once introduced, was clearly recoverable from the context. In speech, the participants showed a more balanced distribution of nouns and verbs, as an overt noun or pronoun must usually be used in BP.

Additionally, all participants used a much higher number of articles, prepositions and conjunctions in speech than in sign. This result is expected, since analogous structures are typically expressed in Libras by the movement of a sign, not by a separate lexical entry. Despite these overall patterns, however, we also noted a rich variability among the categories of words produced in each narrative in both languages for all participants.

The results of our analysis of sentence types (Figure 2) reveal considerable variability among the participants. NT and JB used less complex sentences in Libras than MR and CL did. This finding correlates with the word rates for each language: recall that NT and JB used fewer words per minute compared to MR and CL. Interestingly, NT also used fewer complex sentences when telling the story in Portuguese, while MR used a richer array of sentence types in Libras than in Portuguese, even though she had high word-per-minute rates in both languages. We may speculate that MR adjusted her Portuguese as if telling the story to children, which she did not do when she was signing. As MR is a teacher of children, she may have unconsciously followed this pattern of simpler spoken sentences in this context. CL demonstrated the richest distribution of sentence types in both languages; her word/minute rates were lower than MR’s, but still higher than JB’s and NT’s.

The monolingual comparison reveals again that CL and MR pattern closely to Deaf signers, who make use of a variety of sentence types in their productions. On the other hand, JB and NT contrast with the monolinguals in showing a much reduced variety of sentence types. As for the BP speakers, the variability in their patterning is quite similar to the variability seen across the speech of the bimodal bilinguals, suggesting that the participants are generally comparable in their use of the spoken language.

Table 2 above gives the rates of ungrammatical verbal morphology in the sign of the four bimodal bilinguals. Note that the occurrences of ungrammatical verbal morphology in Libras track the overall fluency level for each participant. Such a pattern is also found in studies of hearing/speaking heritage language users—those who show weaknesses in rate and sentence complexity overall also tend to show errors in verbal morphology (Albirini et al. 2011, 2013; Montrul 2011).

Many of the observed Libras verbal morphology errors are related to the choice of handshape in depicting verbs. Verbs in this language employ particular handshapes to express semantic categorical information and it seems that NT and JB did not always know the right handshape for their categorical choices. Previous research suggests that mastering this morphological aspect is challenging for firstand second-language learners of various sign languages (e.g., (Karnopp 1994) on Libras). An example from NT is given in (1).

1.*DS(pato?-passou-na-frente-do-caminhão)duck?-pass-in-front-of-truck‘The duck went in front of the truck like so.’

In this example, the notation DS refers to depicting signs, and the description within parentheses explains what the sign is conveying, but the individual words connected by hyphens should not be taken to represent separate signs or morphemes (see (Emmorey 2003) for more discussion about depicting signs, also known as classifier constructions). In the production by NT, the handshape for *PATO* (‘duck’) is wrong but the intention behind the sign can be recovered from the story. First, NT signed *BORBOLETA* (‘butterfly’) in place of *PATO* (‘duck’). Then, he used a C handshape, which would be used to represent holding an object, and moved the C handshape in front of the handshape representing the truck. The target handshape would be one indicating animal legs; there are two acceptable versions in Libras, one with only one hand (5 handshape) or one with both hands signing symmetrically (3 handshape). Since NT appeared to lack sufficient vocabulary to access while he was signing, in his storytelling he often did not use signs or relied on signs that were more gesture-like. Conversely, his sister JB knew the signs but she produced another kind of error in Libras involving the use of signing space to represent referents. Signers should use different locations in space for different referents; these spatial loci are used in the systems of pronominals and verb agreement. However, rather than using different loci for different referents, JB put multiple referents in the same location. Interestingly, this same type of error is also observed at the beginning of the sign language acquisition process among Deaf children of Deaf parents (Bellugi et al. 1990).

Another kind of error in Libras verbal morphology concerns adverbial marking, including markers for repetition and emphasis. There are no occurrences of such adverbial morphology associated with verbs in NT’s data, and there are just a few occurrences of this marker in the productions of JB, but there are many occurrences produced by CL and MR. This also may indicate the poverty of the signs produced by NT and JB. In narratives, it is very typical to have adverbial markers in the signed story because the facts include a lot of information that should be marked. See the example of this marker produced by CL in (2). The sign *SUSTO* (‘frighten’) produced by CL is marked with an adverbial so it means ‘frightened very much’.

2.HOMEM SUSTO+PATODS(pato-passar-frente-caminhão)DS(pato-caminhar)man be-afraidduckduck-cross-in-front-of-the-truckduck-walking‘The man was frightened by the duck who was crossing in front of the truck like this.’

### 4.2. General Discussion

The genre of the productions analyzed in this study is narrative, reducing the opportunities for production of certain sentence types such as conditionals, yes/no questions and WH-questions. Several independent reasons may explain why there is variability in the sentence types used by each storyteller, but when we compare sentence complexity with all the other information regarding individual differences in word/minute production speed, MLU, verbal morphology and the use of adverbial modifications, we can see an indication that each participant relies more heavily on one or the other language as primary (even if this is not the dominant language), as has been reported by Benmamoun et al. (2013) for other heritage speakers.

In these heritage signers, it seems that NT and JB present a divergent Libras structure. However, it is interesting that for NT the story told in Portuguese also presents a low production speed, which is different from the others. Also, for MR, Portuguese has a very small variety of sentence types, while in Libras she presented a greater variety of different types of structures. MR has more contact with her deaf parents than with other signers but she may contact other deaf people on special occasions, and deaf children in the school where she works. CL is the most balanced signer/speaker; as she is also a sign language interpreter, it is not surprising that there are such clear differences between her and the others who sign with their families only or primarily. CL has contact with intellectual deaf signers, since she is an interpreter in the academic level, as well as in Deaf associations and in formal organizations, besides her deaf family. These different contexts may influence the signing skills of the four participants.

We now turn our discussion to addressing two overarching themes for considering bimodal bilinguals as heritage language users.

#### 4.2.1. What Determines the Range of Variation within the Heritage Speaker Cohort that We Are Considering? What is the Role of Language Use in a Given Context?

JB and NT had contact only with their Deaf parents, and a few other Deaf people from their parents’ circle of friends. Furthermore, their parents do not have formal education at all. It seems that this lower level of education combined with access only to a more restricted community of signers may have influenced their signing skills.

Another relevant factor is the difference between participants in their professional involvement with Deaf people. This impacts directly in their bilingual status and in the way that they manage both languages. CL is a balanced bilingual and she is trained to use the two languages in different contexts. Moreover, she developed control of the uses of the languages, even in contexts using code-blending (Lillo-Martin et al. 2010, 2014, 2016). The other participants are not interpreters, but MR is a teacher of Deaf children. She has contact with other Deaf people but she usually restricts her contact to the children that she interacts with, in addition to her Deaf parents. This means that while she has access to a greater variety of signers than JB and NT do, she is still somewhat limited in use of Libras, which can have some implications for her signing skills.

#### 4.2.2. What Areas Are Vulnerable in Both Languages and What Areas Are Robust? Why?

In this study, some bilinguals showed weaknesses in the use of Libras verbal morphology. As with spoken heritage languages, agreement marking appears to be particularly affected. In addition to agreement marking, the morphology of classifier or depicting signs—which requires specific handshape morphemes—was also vulnerable.

The weaker signers also showed a tendency to use simple sentences instead of more complex structures (declarative sentences and coordinate sentences with a few or no occurrences of embedded clauses, relative clauses, conditionals, doubling and other types). Such avoidance of embedded structures is also typical of various heritage languages (Benmamoun et al. 2013) and our results indicate that adherence to shallow structure is independent of modality. Deficiencies in syntactic domains can be investigated more systematically in comprehension tasks, and we leave this for further study.

There is some evidence for weaknesses in the spoken language for only one bimodal bilingual—NT—although it should be kept in mind that these weaknesses may be related to the fact that he was not comfortable with the tests and the cameras. In comparison to the other bilinguals and the monolinguals, NT was slower, had a lower MLUw, and used fewer sentence types in BP. The other three participants showed no weaknesses in their spoken language. This variability may be related to the kind of interactions in sign and in speech that the participants experienced while they were growing up.

## 5. Conclusions

This paper presented and analyzed narrative data from four bimodal bilinguals using Libras and Portuguese in Brazil. We have characterized these bimodal bilinguals as heritage speakers of their sign language. Despite this blanket characterization, there is a great degree of variance across our participants, from the more balanced ones to the ones who are more spoken-language dominant. This variation is reflected in their use of lexical material, rate of language production, and other measures.

Those speakers who can be identified as unbalanced bimodal bilinguals, with the sign language as their weaker language, show a number of properties that are typical of heritage speakers in spoken languages. In particular, their rate of production, MLU, and sentence types used are more restricted than those of the more balanced bilinguals; in addition, they have difficulty in managing material related to morphology, especially agreement and classifiers.

Turning now to the roots of variation in language maintenance, for the speakers considered here, their fluency depended on a number of social factors as well as on the amount of input they received in the sign language. In fact, we found that these factors are tightly intertwined—probably more so than they are intertwined in spoken languages—because the Deaf community is so strongly identified with Deaf culture. Relatedly, another relevant factor in language variation across our participants had to do with their professional involvement with Deaf people, since one of the participants is a professional sign language interpreter. This research is the first step in our understanding of language variation in bimodal bilinguals; additional research considers the ways that bimodal bilinguals combine their languages in the code-blending phenomenon, which provides further means to investigate the nature of heritage bimodal bilingualism (de Quadros 2017; Lillo-Martin et al. 2016).

## Figures and Tables

**Table 1.** Characteristics of the participants.

Group	Participant	Interpreter?	Sign Rating *	Speech Rating *
Bimodal bilinguals	CL	yes	7	7
	MR	no	6	7
	JB	no	4	7
	NT	no	3	6
Deaf signers	RM	no	7	n/a
	FR	no	7	n/a
	SD	no	7	n/a
	MS	no	7	n/a
Hearing non-signers	ZE	no	n/a	7
	SZ	no	n/a	7
	AR	no	n/a	7
	VS	no	n/a	7

* Ratings are based on the observation of a native bimodal bilingual.

**Table 2.** Production speed and mean length of utterance in words (MLUw) for sign and speech.

Group	Participant	Sign Words/Minute	Speech Words/Minute	Ratio Speech:Sign	Sign MLUw	Speech MLUw	%Sign VMorph Errors
Bimodal bilinguals	CL	73.84	74.29	1.01	5.50	7.46	0
	MR	86.25	155.60	1.81	3.63	6.40	3
	JB	63.42	126.00	1.99	3.70	6.47	12
	NT	48.15	41.66	0.87	2.54	5.00	59
Deaf signers	RM	129.12	n/a	n/a	7.27	n/a	n/a
	FR	79.81	n/a	n/a	6,68	n/a	n/a
	SD	77.85	n/a	n/a	6,04	n/a	n/a
	MS	100.71	n/a	n/a	5,64	n/a	n/a
Hearing non-signers	ZE	n/a	102.47	n/a	n/a	9.88	n/a
	SZ	n/a	107.14	n/a	n/a	9.00	n/a
	AR	n/a	147.64	n/a	n/a	8.96	n/a
	VS	n/a	143.63	n/a	n/a	6.37	n/a

## Footnotes

1 We use a capital D for Deaf to indicate the linguistic-cultural group rather than a medical diagnosis.

2 We call the spoken language the second language because of chronology, even though the spoken language ends up being the dominant language (or the other language if they are balanced bilinguals).

3 Research approval was granted by the Review Board at the Universidade Federal de Santa Catarina. IRB: CAAE 0014.0.242.000-09.

## References

[R1] Albirini Abdulkafi, Benmamoun Elabbas, and Saadah Eman. 2011. Grammatical features of Egyptian and Palestinian Arabic heritage speakers’ oral production. Studies in Second Language Acquisition 33: 273–303.

[R2] Albirini Abdulkafi, Benmamoun Elabbas, and Brahim Chakrani. 2013. Gender and number agreement in the oral production of Arabic Heritage speakers. Bilingualism: Language and Cognition 16: 1–18.

[R3] Bellugi Ursula, Lillo-Martin Diane, O’Grady Lucinda, and van Hoeck Karen. 1990. The Development of Spatialized Syntatic Mechanisms in American Sign Language. In The Fourth International Sympsium on Sign Language Research. Edited by Edmondson William H. and Karlson Fred. Hamburg: Signum Verlag Press, pp. 16–25.

[R4] Benmamoun Elabbas, Montrul Silvina, and Polinsky Maria. 2013. Heritage languages and their speakers: Opportunities and challenges for linguistics. Theoretical Linguistics 39: 129–81.

[R5] Chomsky Noam. 1986. Knowledge of Language: Its Origin, Nature and Use. New York: Praeger.

[R6] Compton Sarah E. 2014. American Sign Language as a Heritage Language. In Handbook of Heritage, Community, and Native American Languages in the United States: Research, Policy, and Educational Practice. Edited by Wiley Terrence G., Peyton Joy Kreeft, Christian Donna, Moore Sarah Catherine K. and Liu Na. New York: Routledge, New York: Center for Applied Linguistics.

[R7] Crasborn Onno, and Sloetjes Han. 2008. Enhanced ELAN functionality for sign language corpora. In Construction and Exploitation of Sign Language Corpora. 3rd Workshop on the Representation and Processing of Sign Languages. Edited by Crasborn O, Efthimiou E, Hanke T, Thoutenhoofd E and Zwitserlood I. Parijs: ELDA, pp. 39–43.

[R8] De Quadros Ronice Müller. 1995. As Categorias Vazias Pronominais. Master dissertation, Pontifícia Universidade Católica do Rio Grande do Sul, Porto Alegre, Brazil.

[R9] De Quadros Ronice Müller. 2017. Língua de herança: Língua brasileira de sinais. Porto Alegre: Penso Editora.

[R10] De Quadros Ronice Müller, Lillo-Martin Diane, and Emmorey Karen. 2016a. As línguas de bilíngues bimodais. Revista de Estudos Linguísticos da Univerdade do Porto 11: 139–60.

[R11] de Quadros Ronice Müller, Lillo-Martin Diane, and Pichler Deborah Chen. 2016b. Bimodal Bilingualism: Sign Language and Spoken Language. In The Oxford Handbook of Deaf Studies in Language. Edited by Marschark Marc and Spencer Patricia Elisabeth. Oxford: Oxford University Press, pp. 181–96.

[R12] Döpke Susanne. 1992. One Parent—One Language: An Interactional Approach. Amsterdam: John Benjamins Publishing Company.

[R13] Emmorey Karen. 2003. Perspectives on Classifier Constructions in Sign Languages. Mahwah: Lawrence Erlbaum Associates.

[R14] Emmorey Karen, Borinstein Helsa B., Thompson Robin, and Gollan Tamar H.. 2008. Bimodal bilingualism. Bilingualism: Language and Cognition 11: 43–61. 19079743 10.1017/S1366728907003203 PMC2600850

[R15] Fishman Joshua. 2001. 300-plus years of heritage language education in the United States. In Heritage Languages in America: Preserving a National Resource. Edited by Peyton Joy Kreeft, Ranard Donald A. and McGinnis Scott. Washington, DC and McHenry: Center for Applied Linguistics and Delta Systems, pp. 81–89.

[R16] Johnston Trevor. 1991. Transcription and glossing of sign language texts: examples from AUSLAN (Australian Sign Language). International Journal of Sign Linguistics 2: 3–28.

[R17] Kanto Laura, Huttunen Kerttu, and Laakso Marja-Leena. 2013. Relationship between the linguistic environments and early bilingual language development of hearing children in deaf-parented families. Journal of Deaf Studies and Deaf Education 18: 242–60. 23349396 10.1093/deafed/ens071

[R18] Karnopp Lodenir Becker. 1994. Aquisição do Parâmetro Configuração de Mão dos Sinais da LIBRAS: estudo sobre quatro crianças surdas filhas de pais surdos. Ph.D. dissertation, Instituto de Letras e Artes, Pontifícia Universidade Católica do Rio Grande do Sul, Porto Alegre, Brazil.

[R19] Kondo-Brown Kimi, ed. 2006. Heritage Language Development: Focus on East Asian Immigrants. Amsterdam: John Benjamins.

[R20] Lanza Elisabeth. 1997. Language Mixing in Infant Bilingualism. New York: Oxford University Press.

[R21] Lillo-Martin Diane, de Quadros Ronice Müller, Koulidobrova Elena, and Pichler Deborah Chen. 2010. Bimodal bilingual cross-language influence in unexpected domains. In Language Acquisition and Development: Proceedings of GALA 2009. Edited by Costa João, Castro Ana, Lobo Maria and Pratas Fernanda. Newcastle upon Tyne: Cambridge Scholars Press, pp. 264–75.

[R22] Lillo-Martin Diane, de Quadros Ronice Müller, Pichler Deborah Chen, and Fieldsteel Zoe. 2014. Language choice in bimodal bilingual development. Frontiers in Psychology 5: 1163. 25368591 10.3389/fpsyg.2014.01163 PMC4202712

[R23] Lillo-Martin Diane, de Quadros Ronice Müller, and Pichler Deborah Chen. 2016. The development of bimodal bilingualism: Implications for linguistic theory. Linguistic Approaches to Bilingualism 6: 719–55. 28603576 10.1075/lab.6.6.01lil PMC5461974

[R24] Montrul Silvina. 2011. Morphological errors in Spanish second language learners and heritage speakers. Studies in Second Language Acquisition 33: 163–92.

[R25] Palmer Jeffrey Levi. 2015. ASL Word Order Development in Bimodal Bilingual Children: Early Syntax of Hearing and Cochlear-Implanted Deaf Children From Signing Families. Ph.D. dissertation, Gallaudet University, Washington, DC, USA.

[R26] Petroj Vanessa, Guerrera Katelyn, and Davidson Kathryn. 2014. ASL Dominant Code Blending in the Whispering of Bimodal Bilingual Children. In BUCLD 38: Proceedings of the 38th annual Boston University Conference on Language Development. Edited by Orman Will and Valleau Matthew James. Somerville: Cascadilla Press, vol. 2, pp. 319–30.

[R27] Peyton Joy Kreeft, Ranard Donald A., and McGinnis Scott, eds. 2001. Heritage Languages in America: Preserving a National Resource. Washington, DC and McHenry: Center for Applied Linguistics and Delta Systems.

[R28] Pichler Deborah C., Hochgesang Julie, Lillo-Martin Diane, and de Quadros Ronice Müller. 2010. Conventions for sign and speech transcription in child bimodal bilingual corpora in ELAN. Language, Interaction and Acquisition 1: 11–40. 10.1075/lia.1.1.03che PMC3102315 21625371

[R29] Pichler Deborah C., Lee James, and Lillo-Martin Diane. 2014. Language development in ASL-English bimodal bilinguals. In Multilingual Aspects of Signed Language Communication and Disorder. Edited by Quinto-Pozos David. Bristol: Multilingual Matters, pp. 235–60.

[R30] Pichler Deborah C., Reynolds Wanette, Palmer Jeffrey Levi, de Quadros Ronice Müller, Kozak Viola Laura, and Lillo-Martin Diane. 2017. Heritage signers: Bimodal bilingual children from deaf families. In Language Acquisition at the Interfaces: Proceedings of GALA 2015. Edited by Choi Jiyoung, Demirdache Hamida, Lungu Oana and Voeltzel Laurence. Newcastle upon Tyne: Cambridge Scholars Publishing, pp. 247–69.

[R31] Pizer Ginger Bianca. 2008. Sign and Speech in Family Interaction: Language Choices of Deaf Parents and their Hearing Children. Ph.D. dissertation, The University of Texas at Austin, Austin, TX, USA.

[R32] Polinsky Maria. 2005. Word class distinctions in an incomplete grammar. In Perspectives on Language and Language Development. Edited by Polinsky Maria, Ravid Dorit and Shyldkrot Hava Bat-Zeev. Dordrecht: Kluwer Academic Press, pp. 419–36.

[R33] Polinsky Maria. 2006. Incomplete acquisition: American Russian. Journal of Slavic Linguistics 14: 191–262.

[R34] Polinsky Maria. 2008. Gender under Incomplete Acquisition: Heritage Speakers’ Knowledge of Noun Categorization. Heritage Language Journal 6: 40–71.

[R35] Pyers Jennie E., and Emmorey Karen. 2008. The face of bimodal bilingualism: Grammatical markers in American Sign Language are produced when bilinguals speak to English monolinguals. Psychological Science 19: 531–35. 18578841 10.1111/j.1467-9280.2008.02119.x PMC2632943

[R36] Reynolds Wanette. 2016. Early Bimodal Bilingual Development of ASL Narrative Referent Cohesion: Using a Heritage Language Framework. Ph.D. dissertation, Gallaudet University, Washington, DC, USA.

[R37] Scontras Gregory, Fuchs Zuzanna, and Polinsky Maria. 2015. Heritage language and linguistic theory. Frontiers in Psychology 6: 1545. 26500595 10.3389/fpsyg.2015.01545 PMC4598584

[R38] van den Bogaerde Beppie, and Baker Anne E.. 2009. Bimodal language acquisition in Kodas. In Hearing, Mother-Father Deaf: Hearing People in Deaf Families. Edited by Bishop Michele and Hicks Sherry L.. Washington, DC: Gallaudet University Press, pp. 99–131.

